# Dynamic Compressive Damage Constitutive Correction of Concrete Under Freeze-Thaw Cycle

**DOI:** 10.3390/ma18061238

**Published:** 2025-03-11

**Authors:** Ankui Hu, Xinglin Chen, Xinyu Du, Fei Wang

**Affiliations:** 1 Key Laboratory of Fluid and Power Machinery, Xihua University, Ministry of Education, Chengdu 610039, China; feiwang201268@163.com; 2 Key Laboratory of Fluid Machinery and Engineering, Xihua University, Chengdu 610039, China; 3 School of Energy and Power Engineering, Xihua University, Chengdu 610039, China; cxl200338@163.com (X.C.); dxy20010429@163.com (X.D.)

**Keywords:** concrete, freeze-thaw cycles, damage modification, finite element simulation

## Abstract

In order to investigate the uniaxial dynamic compression constitutive model for concrete under freeze-thaw conditions, a finite element analysis was employed to model the temperature field of the concrete. The resulting data were subsequently integrated into the thermal stress field as a predefined parameter for a sequentially coupled thermal stress analysis. Following this, a numerical simulation was performed to assess concrete damage progression during a dynamic compression test in a freeze-thaw setting. The constitutive model was developed by adjusting the parameters that govern the development of concrete damage within the uniaxial stress-strain relationship, as specified in the Code for Design of Concrete Structures. The uniaxial compression behavior of concrete subjected to freeze-thaw was simulated utilizing both the modified intrinsic model and the damage plasticity model available in the software while accounting for the effects of the damage factor. The findings indicate that the modified constitutive model aligns closely with the actual experimental results. These research outcomes have potential applications in numerical simulations and various engineering practices related to concrete.

## 1. Introduction

Concrete structures are crucial in modern civil engineering, especially in China’s infrastructure development. With the widespread use of concrete dams, particularly the over 100,000 reservoir dams mostly composed of high concrete, their operational environments have become more complex. As these dams age, material deterioration becomes apparent, impacting their long-term safety. In extreme cases, this can lead to dam failure, posing significant risks to downstream populations and property [1]. In the cold northern regions, numerous hydraulic structures encounter complex stress states, and the freeze-thaw cycles can negatively impact the physical characteristics of hydraulic concrete. The Fengman Dam is located in Jilin Province, where the lowest daily average temperature reaches −30.7 °C, and the instantaneous lowest temperature drops to −40.5 °C. During a quality inspection in 1963, the non-overflow dam section on the downstream face suffered from freeze-thaw damage covering an area of 6600 square meters, with severe concrete spalling and damage depths ranging from 10 to 40 cm, and in severe areas, up to 1 to 2 m. The Yunfeng Dam experiences a lowest daily average temperature of −32.6 °C. During a quality inspection in 1980, the non-overflow dam section on its downstream face exhibited freeze-thaw damage over an area of 9217 square meters, with layered concrete spalling and damage depths of 3 to 15 cm [2]. Therefore, it is imperative to investigate the physical characteristics of these structures under complex stress conditions following freeze-thaw cycles [3].

Many studies have been carried out on the characteristics of concrete after it has undergone freeze-thaw damage. Demir et al. [4] investigated the effect of freeze-thaw cycles on the performance of concrete by studying cement composites incorporated with optimized Class F and Class C fly ashes (FA). Tian et al. [5] examined the mechanical characteristics of concrete specimens subjected to freeze-thaw cycles and axial fatigue loading, utilizing compressive strength as a primary metric. Their study involved a comparative analysis of the alterations in compressive properties before and after exposure to these conditions, as well as an investigation into the progression of mechanical property degradation resulting from freeze-thaw cycles and axial fatigue. Xu et al. [6] integrated the effects of freeze-thaw cycles with salt ion erosion, exploring variations in the early cubic compressive strength, relative dynamic modulus of elasticity, mass loss rate, and thickness of the damaged layer while also investigating the underlying damage mechanisms through controlled rapid freeze-thaw tests. Long et al. [7] developed a uniaxial compression constitutive model to investigate the stress-strain relationships of C40 normal concrete (NC) and self-compacting concrete (SCC) following repeated freezing and thawing processes. Currently, the majority of research has focused on the mechanical characteristics and damage evolution of modified concrete [8,9,10,11,12,13,14,15,16,17,18,19]. However, research on the compressive performance of concrete still primarily relies on static macroscopic testing. Some dynamic studies have only considered the mechanical properties at the macroscopic level, with limited analysis on the evolution of internal damage under dynamic loads that may occur in actual engineering projects. Currently, there is an urgent need to supplement the simulations of the dynamic constitutive relationship of concrete under freeze-thaw cycles.

**Figure 1:**
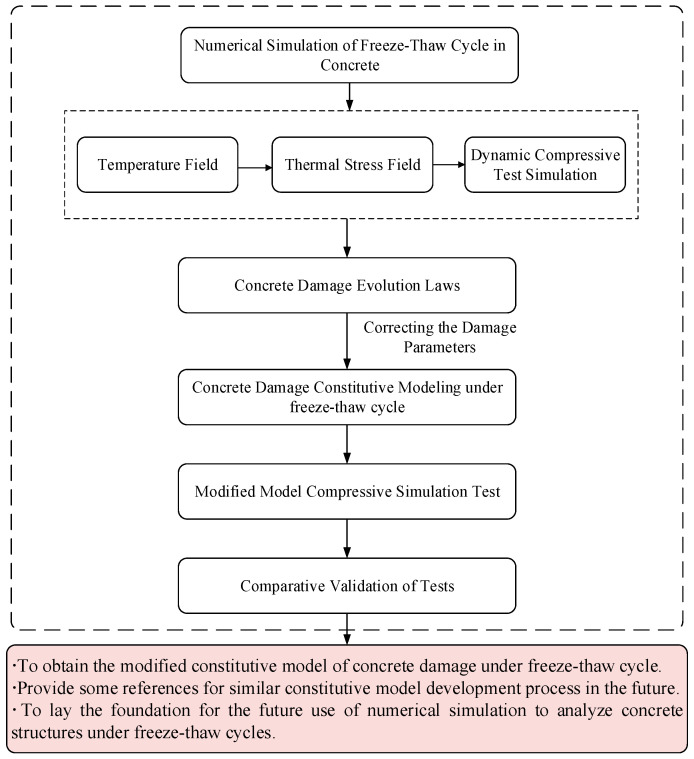
Technology Roadmap.

To address this research gap, this study employs a combination of experimental and numerical methods. We use the large-scale finite element software ABAQUS 2023 to analyze heat transfer in concrete specimens during freeze-thaw cycles. Then, through explicit dynamic analysis (ABAQUS/Explicit [20]), we experimentally study the uniaxial compressive strength of these specimens at a strain rate of 10^–2^/s after different numbers of freeze-thaw cycles. Our goal is to summarize the mechanical performance degradation patterns of concrete structures. Based on these patterns, we revise the concrete damage evolution parameters in the uniaxial compressive stress-strain curve for ordinary concrete in the current Code for Design of Concrete Structures [21] (referred to as the *Code*). This leads to a new constitutive model for concrete damage under freeze-thaw cycles. Since this model is derived from the constitutive model in the *Code* with modifications, the applicable concrete must meet the following specific criteria: the maximum water-binder ratio should not exceed 0.55, the chloride ion content should not exceed 0.15%, the alkali content should not exceed 3.0 kg/m^3^, the strength grade should range from C20 to C80, and the mass density should be between 2200 kg/m^3^ and 2400 kg/m^3^. We validate the revised model by comparing its results with experimental data. Details are shown in Figure 1. This work provides theoretical and practical guidance for analyzing the strength and deformation of concrete under complex stresses after freeze-thaw cycles.

## 2. Calculation Model and Analysis Results

This study employs both static and dynamic analytical methods to delineate the evolution of uniaxial compressive damage in concrete subjected to freeze-thaw cycles. The numerical simulation of the freeze-thaw process is executed through a sequential coupling of thermal stress analysis. The procedure commences with an analysis of thermal conduction, the results of which are subsequently incorporated into the static analysis phase. Thereafter, the outcomes from the static analysis are utilized as initial conditions for the dynamic explicit analysis, during which dynamic loading is assessed.

### 2.1. Static Step Analysis Model

The model employed in this study consists of a prismatic test block with dimensions of 150 mm × 150 mm × 300 mm. The parameters for analysis are configured to assess heat transfer, with material properties and intrinsic curves derived from the design specifications outlined in the *Code* [21]. The concrete strength grade is classified as C30. The ambient temperature is set at 1 °C, and the temperature amplitude is adjusted to facilitate alternating temperature cycles ranging from −20 °C to 10 °C, thereby simulating various freeze-thaw cycles. The material parameters utilized are detailed in Table 1. According to the “Standard for Test Methods of Long-term Performance and Durability of Ordinary Concrete” (GB/T 50082–2009) [22], using the “quick-freezing method”. Each freeze–thaw cycle should be completed within 2–4 h, and the time for melting should not be less than 1/4 of the whole freeze–thaw cycle time. The conversion time between freezing and thawing should not exceed 10 minutes. We considered one freeze-thaw cycle as a period of 4 h. Consequently, the corresponding durations for 25, 50, 75, and 100 freeze-thaw cycles are calculated to be 3.6 × 10^5^ s, 7.2 × 10^5^ s, 1.08 × 10^6^ s, and 1.44 × 10^6^ s, respectively. For the specific settings of temperature amplitude within one cycle, please refer to Table 2. The duration for freezing is 2.25 h, and the duration for thawing is 1.75 h. Upon the completion of the temperature field analysis, the analysis step is configured to “Static, General”, and the unit type is modified to 3D Stress. The results from the temperature field analysis are imported as a predefined field, while all other settings remain unchanged.

**Figure 2:**
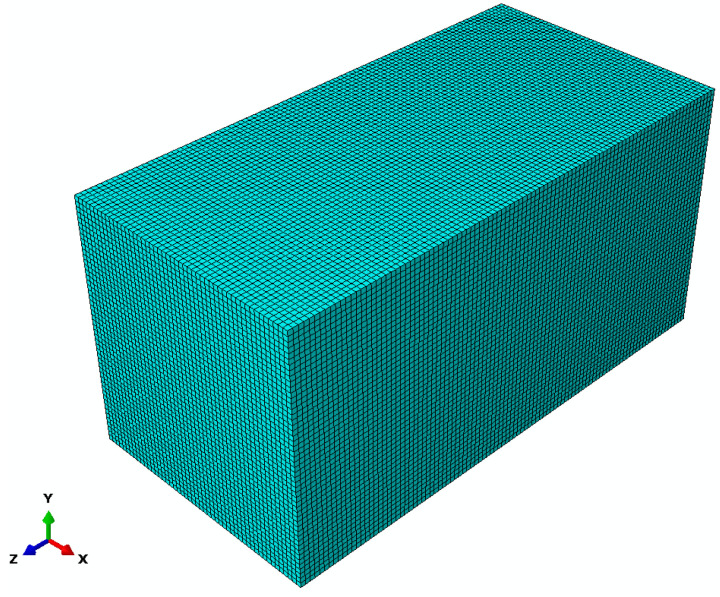
Concrete Test Block Model Diagram.

Shown in Figure 2 is the model diagram of the concrete structure.

### 2.2. Heat Transfer Analysis Results

**Figure 3:**
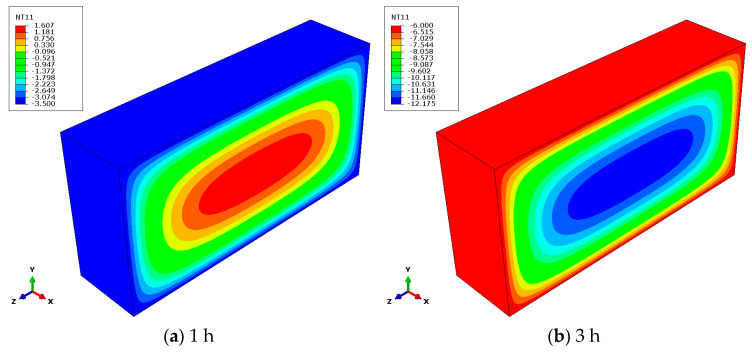
Temperature change cloud map of the first freeze-thaw cycle.

Upon the completion of the heat conduction analysis, the temperature variation cloud diagram is analyzed, as depicted in Figure 3. The data indicate that the internal temperature distribution within the concrete exhibits a concentric ring pattern, with the cooling process characterized by a sequential reduction from the interior to the exterior. This phenomenon suggests that the external temperature propagates inward in layers, resulting in a temporal lag of the internal temperature in relation to the external temperature, thereby creating a temperature differential between the interior and exterior. Following multiple freeze-thaw cycles, the cumulative effect of the temperature disparity imposes repeated thermal stress on the specimen, ultimately leading to freeze-thaw damage.

**Figure 4:**
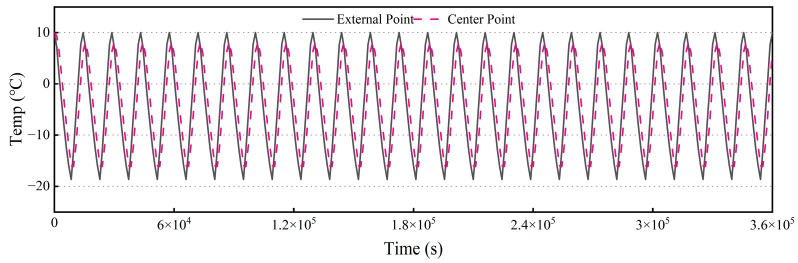
Temperature change curve over 25 freeze-thaw cycles.

To conduct a comprehensive analysis of this phenomenon, an external point relative to the concrete is selected, and the temperature variation curve of a specific node over time is plotted, utilizing 25 freeze-thaw cycles as a case study, as illustrated in Figure 4. The data reveal that the temperature at the node fluctuates between approximately −20 °C and 10 °C, demonstrating a cyclic pattern with a duration of 4 h per cycle, which is consistent with the temperature range established by the rapid freeze-thaw apparatus. Subsequently, the temperature change curve at the center of the concrete specimen is analyzed, indicating that the temperature at this central point lags behind the temperature changes observed at the surface. The minimum temperature recorded at the center of the specimen is −16.15 °C, while the maximum temperature reaches 8.18 °C, aligning with the stipulated criteria for the freeze-thaw testing protocol. Specifically, at the conclusion of the cooling phase, the center temperature of the specimen should be maintained within the range of −18 ± 2 °C, and at the conclusion of the heating phase, it should be controlled at 5 ± 2 °C.

### 2.3. Dynamic Step Analysis Model

**Figure 5:**
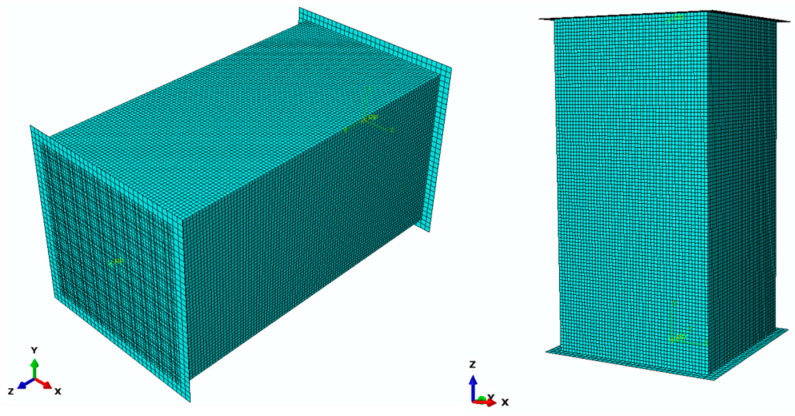
Dynamic Step Analysis Model Diagram.

As shown in Figure 5, the specimen model grid and the dimensions of the static step model are congruent, with the assembly at both ends of the specimen measuring 170 mm × 170 mm and incorporating rigid body pads. General contact is established between the pads and the surface of the specimen, with tangential behavior characterized by a penalty friction model, while the normal contact is defined as hard contact. The loading rate is set at 3 mm/s, corresponding to a specimen length of 300 mm, which yields a strain rate of 10^−2^/s. All constitutive relations are based on C30 concrete, as delineated in the *Code* [21].

### 2.4. Dynamic Load Analysis Results

A controlled displacement was applied to the specimens, and the reaction force was monitored at the load application points. The displacement was gradually increased until failure occurred, at which point the maximum force (corresponding to the peak load) was recorded. This value represents the maximum compressive load that the specimen could withstand before failure. To ensure the accuracy and reliability of the results, multiple simulations were conducted for each specimen, and the average maximum force was calculated.

**Figure 6:**
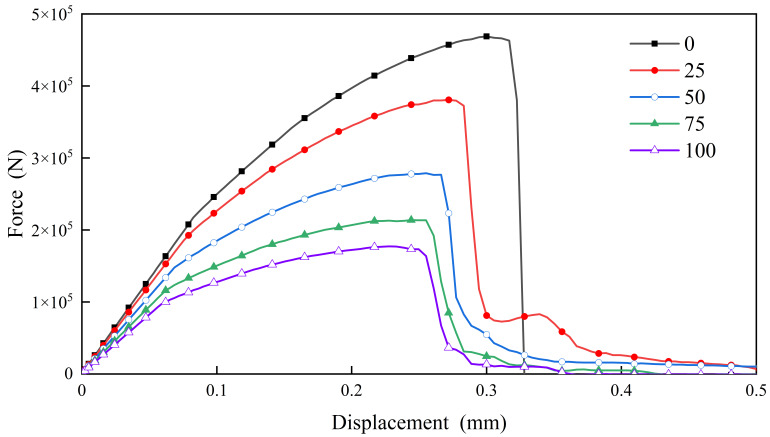
Force-displacement curves at 10^−2^/s strain rate.

The test data have been systematically organized and analyzed, revealing the definitive force-displacement curves for C30 concrete subjected to varying numbers of freeze-thaw cycles at a strain rate of 10^–2^/s, as illustrated in Figure 6 below.

The figure demonstrates that the morphology of the stress-strain curve exhibits a consistent trend across different counts of freeze-thaw cycles. Specifically, as the number of freeze-thaw cycles increases, there is a continuous decline in both the peak load and the corresponding peak displacement. This phenomenon can be attributed to the degradation of mechanical characteristics and the proliferation of microcracks, both on the surface and within the concrete matrix, resulting from freeze-thaw cycles, which ultimately results in a reduction of the peak displacement and peak load of the concrete.

After collating the test data, the compressive strength was calculated according to Equation (1).

(1) $$f_{c}=\frac{F}{A}$$

In this context, $f_{c}$ is the compressive strength of concrete prismatic specimens, MPa, accurate to 0.01 MPa; F is the specimen breaking force, N; A is the specimen pressure-bearing area, mm^2^.

The results after collating the data are shown in Table 3.

To deeply explore the quantitative relationship between the compressive strength of concrete and the number of freeze-thaw cycles, this study employed a Logistic regression model to fit the data presented in Table 3. The Logistic model was selected as the analytical tool for this study due to its ability to describe the characteristic changes of the dependent variable within a certain range, especially demonstrating good applicability when approaching extreme values. By conducting Logistic regression analysis on these data, we obtained a highly fitted model with a coefficient of determination R² reaching 0.9996, indicating that the model can excellently explain the variation in compressive strength of concrete with the number of freeze-thaw cycles. The expression of the model is as follows:

(2) $$f_{c}^{D}/f_{c}=\frac{0.795}{1+{\left(N/49.59\right)}^{1.89}}+0.205\;(R^{2}=0.9996)$$

In this context, $f_{c}^{D}$ is the compressive strength of the concrete after freeze-thaw; $f_{c}$ is the compressive strength of the concrete before freeze-thaw; N is the number of freeze-thaw cycles.

Table 3 presents the degradation pattern of concrete compressive strength resulting from freeze-thaw cycles. After enduring 100 freeze-thaw cycles, the compressive strength of the concrete is measured at 7.7 MPa, indicating a reduction of 63.03% in comparison to its strength under non-freeze-thaw conditions. During the initial phase of the freeze-thaw cycles, the uneven distribution of internal aggregates and moisture contributes to rapid spalling due to freeze-thaw damage, which results in the formation of microcracks and a distinct damage layer of considerable thickness. Consequently, a significant decline in strength is observed. As the number of freeze-thaw cycles increases, the rate of strength reduction diminishes slightly; however, the thickness of the damaged layer continues to increase, leading to the ongoing deterioration of the concrete. This deterioration ultimately fails to meet engineering standards and may result in complete structural failure. Prior research [15,17] categorizes the damage to concrete under freeze-thaw cycles into three distinct phases: rapid growth, slow development, and damage. The first phase is characterized by an accelerated rate of damage accumulation, resulting in significant and irreversible harm to the concrete. The subsequent phase, referred to as the slow development stage, experiences a continued increase in damage, albeit at a reduced rate compared to the initial phase. The final phase, referred to as the damage stage, is marked by the complete destruction of the concrete, with the emergence of visible and penetrating cracks, leading to a significantly compromised matrix. This progression closely aligns with the strength variation patterns illustrated in Table 3.

## 3. Concrete Constitutive Modeling Under Freeze-Thaw Cycles

In this section, we have referenced the constitutive empirical equations for concrete provided in the *Code* [21] and have made necessary modifications to better suit the specific concrete materials and loading conditions in our study. The concrete constitutive empirical equations referenced in our paper are based on extensive experimental data and theoretical analysis, which have been widely recognized and adopted in the field of concrete structure design. These equations capture the mechanical behavior of concrete under various loading conditions, including its stress-strain relationship, strength characteristics, and deformation properties. The basic principles behind these equations are rooted in the material science of concrete, taking into account factors such as the composition, mixing ratio, and curing conditions of the concrete.

### 3.1. Changing Law of the Dynamic Modulus of Elasticity of Concrete

The dynamic modulus of elasticity is defined as the ratio of stress to strain in a material when subjected to dynamic loading. This property is typically evaluated using dynamic methods that measure the velocity of elastic wave propagation within concrete. The dynamic modulus serves as an external indicator of the internal damage present in the concrete, which is intrinsically related to the material’s structural characteristics. As internal damage progresses within the concrete, variations in its dynamic elastic modulus can be observed. Guo [23] conducted an experimental investigation into the dynamic elastic modulus of concrete subjected to varying numbers of freeze-thaw cycles, with the detailed findings presented in Table 4.

To deeply explore the quantitative relationship between the dynamic elastic modulus of concrete and the number of freeze-thaw cycles, Logistic regression analysis was performed on the data presented in Table 4. We obtained a highly fitted model with a coefficient of determination R² reaching 0.9879, indicating that the model can well explain the variation in the dynamic elastic modulus of concrete with the number of freeze-thaw cycles. The relationship between the dynamic elastic modulus of concrete and the number of freeze-thaw cycles can be expressed as:

(3) $$E_{0}^{D}/E_{0}=\frac{0.675}{1+{\left(N/87.64\right)}^{1.77}}+0.325$$

In this context, $E_{0}^{D}$ is the dynamic modulus of the concrete after freeze-thaw; $E_{0}$ is the dynamic modulus of the concrete before freeze-thaw; N is the number of freeze-thaw cycles.

### 3.2. Concrete Compressive Damage Evolution Parameters

Compressive strength is a fundamental mechanical property that significantly influences the macroscopic characteristics of concrete, thereby impacting the overall performance of concrete specimens. It is commonly used as a criterion for assessing the durability of concrete structures.

By applying Equations (2) and (3) in conjunction with the uniaxial compressive dc-x curve of standard concrete [21], one can derive the equation that represents the uniaxial compressive dc-x curve of concrete exposed to freeze-thaw cycles. Specifically, this involves substituting the dynamic elastic modulus ($E_{0}$) and axial compressive strength ($f_{c}$) in the parameters: $\varepsilon_{c,r}$, $\rho_{c}$, and n, with their respective counterparts, $E_{0}$ and $f_{c}^{D}$, after exposure to freeze-thaw damage. This adjustment accounts for the deterioration in mechanical properties due to freeze-thaw cycling, thereby providing a more accurate representation of the concrete’s behavior under such conditions. By incorporating the effects of freeze-thaw cycling into the dc-x curve equation, researchers can gain a deeper understanding of the durability and long-term performance of concrete structures subjected to such environmental conditions.

(4) $$\left\{\begin{array}{l} d_{c}=\left\{\begin{array}{cc} 1-\frac{\rho_{c}n}{n-1+x^{n}} & x\leq 1\\ 1-\frac{\rho_{c}}{\alpha_{c}{\left(x-1\right)}^{2}+x} & x>1 \end{array}\right.\\ \rho_{c}=\frac{f_{c}^{D}}{E_{0}^{D}\varepsilon_{c,r}}\\ \begin{array}{l} n=\frac{E_{0}^{D}\varepsilon_{c,r}}{E_{0}^{D}\varepsilon_{c,r}-f_{c}^{D}}\\ \varepsilon_{c,r}=\left(700+172\sqrt{f_{c}^{D}}\right)\times 10^{-6} \end{array}\\ \alpha_{c}=0.157{\left(f_{c}\right)}^{0.785}-0.905 \end{array}\right.$$

In this context, $d_{c}$ is the concrete uniaxial compressive damage evolution parameter; $\varepsilon_{c,r}$ is the strain corresponding to the peak compressive stress; $\rho_{c}$ is the ratio of the concrete uniaxial compressive peak stress to the concrete elastic state peak compressive stress; *n* is the ratio of ${E_{0}^{D}\varepsilon }_{c,r}$ (the peak compressive stress in the elastic state of concrete) to ${E_{0}^{D}\varepsilon }_{c,r}-f_{c}^{D}$ (difference between the elastic state peak compressive stress and the uniaxial peak compressive stress); $\alpha_{c}$ is the value of the parameter of the descending section of the concrete uniaxial compressive stress-strain curve; x is the ratio of the concrete compressive strain to the peak strain.

### 3.3. Concrete Constitutive Modeling

By substituting Equation (4) into the uniaxial stress-strain constitutive relationship for concrete, $\sigma =\left(1-d\right)E_{0}^{D}\varepsilon$, it is possible to derive the uniaxial dynamic compressive stress-strain constitutive model for concrete that is exposed to freeze-thaw cycles.

(5) $$\sigma_{c}=\left\{\begin{array}{cc} \frac{\rho_{c}n}{n-1+x_{c}^{n}}E_{0}^{D}\varepsilon_{c} & x_{c}\leq 1\\ \frac{\rho_{c}}{\alpha_{c}{\left(x_{c}-1\right)}^{2}+x_{c}}E_{0}^{D}\varepsilon_{c} & x_{c}>1 \end{array}\right.$$

The tensile constitutive relationship is established based on the uniaxial tensile stress-strain curve for plain concrete, as outlined in the *Code* [21].

(6) $$\left\{\begin{array}{l} \sigma_{t}=\left\{\begin{array}{cc} \rho_{t}\left(1.2-0.2x_{t}^{5}\right)E_{0}^{D}\varepsilon_{t} & x_{t}\leq 1\\ \frac{\rho_{t}}{\alpha_{t}{\left(x_{t}-1\right)}^{1.7}+x_{t}}E_{0}^{D}\varepsilon_{t} & x_{t}>1 \end{array}\right.\\ \rho_{t}=\frac{f_{t}}{E_{0}^{D}\varepsilon_{t,r}}\\ \varepsilon_{t,r}=65{\left(f_{t}\right)}^{0.54}\times 10^{-6}\\ \alpha_{t}=0.312{\left(f_{t}\right)}^{2}\\ f_{t}=0.395{\left(f_{c}^{D}\right)}^{0.55} \end{array}\right.$$

(7) $$\left\{\begin{array}{c} \varepsilon_{c}=x_{c}\varepsilon_{c,r}\\ \varepsilon_{t}=x_{t}\varepsilon_{t,r} \end{array}\right.$$

The application of the aforementioned constitutive model allows for the determination of the stress-strain relationship of concrete following freeze-thaw damage.

### 3.4. Concrete Damage Plasticity Modeling

The concrete constitutive equations delineated in the *Code* [21] are exclusively focused on elastic strains, and the parameters that govern damage evolution do not align with the damage factors employed in the ABAQUS Concrete Plastic Damage model, which incorporates plastic strains. The Concrete Plastic Damage model in ABAQUS exhibits several defining characteristics [24]: (1) it is a continuous, plasticity-based model for concrete damage; (2) it utilizes anisotropic elastic damage in conjunction with isotropic tensile and compressive plasticity theories to effectively capture the inelastic behavior of concrete; and (3) it recognizes tensile cracking and compressive crushing as the primary mechanisms of damage in concrete. The software requires the input of real stress and real strain to accurately characterize the plasticity of concrete, whereas experimental tests are conducted using nominal stress and strain. Therefore, a conversion from nominal stress and strain to actual stress and strain is necessary, as outlined below:

(8) $$\left\{\begin{array}{l} \sigma_{\mathrm{true}}=\sigma_{n}\left(1+\varepsilon_{n}\right)\\ \varepsilon_{\mathrm{true}}=\ln\left(1+\varepsilon_{n}\right) \end{array}\right.$$

In the compression zone, the damage plasticity model effectively characterizes the degradation of material properties and the corresponding reduction in stiffness by analyzing the relationship between stress and inelastic strain $\left(\sigma -\varepsilon_{c}^{in}\right)$. Additionally, it elucidates the interrelationship among compressive stress, strain, and inelastic strain in concrete.

(9) $$\varepsilon_{c}^{\mathrm{in}}=\varepsilon_{c,\mathrm{true}}-\frac{\sigma_{c,\mathrm{true}}}{E_{0}}$$

In this context, $\varepsilon_{c}^{in}$ is the inelastic strain; $\varepsilon_{t,true}$ is the true compressive strain; $\sigma_{c,true}$ is the true compressive stress.

In the tensile region, the damage plasticity model effectively characterizes material degradation and the reduction of stiffness by analyzing the relationship between stress and cracking strain. This model elucidates the correlation between tensile stress and strain in concrete, along with the corresponding cracking strain.

(10) $$\varepsilon_{c}^{\mathrm{ck}}=\varepsilon_{t,\mathrm{true}}-\frac{\sigma_{t,\mathrm{true}}}{E_{0}}$$

In this context, $\varepsilon_{c}^{ck}$ is the cracking strain; $\varepsilon_{t,true}$ is the true tensile strain; $\sigma_{t,true}$ is the true tensile stress.

In the current study, the Sidoroff energy-equivalent damage model [25,26] was utilized to ascertain the parameters of compressive damage and tensile damage in concrete, employing the following equations:

(11) $$\left\{\begin{array}{l} D_{c}=1-\sqrt{\frac{\sigma_{c,\mathrm{true}}}{E_{0}\varepsilon_{c,\mathrm{true}}}}\\ D_{t}=1-\sqrt{\frac{\sigma_{t,\mathrm{true}}}{E_{0}\varepsilon_{t,\mathrm{true}}}} \end{array}\right.$$

In this context, $D_{c}$ is the concrete compressive damage factor; $D_{t}$ is the concrete tensile damage factor.

## 4. Discussion

### 4.1. Finite Element Simulation Results

The numerical simulation analysis was conducted using the previously established constitutive model.

The compressive damage observed in a concrete prism is fundamentally characterized by the initiation and propagation of microcracks within the specimen, ultimately resulting in a state of penetrating failure. Initially, when the specimen is subjected to loading stress, the strain exhibits a nearly proportional relationship to the growth of these microcracks. As the load continues to escalate, the compressive stress within the test block rises, facilitating the development of plastic deformation and microcracks in the concrete, which in turn leads to a gradual acceleration in strain growth. When the stress in the test block approaches 80% to 90% of the peak stress, the volume compression deformation reaches its maximum value and ceases to decrease further. At this point, the internal microcracks within the concrete exhibit significant development, although no visible cracks are apparent on the specimen’s surface, resulting in a rapid increase in the deformation of the concrete specimen. Upon reaching the peak stress, any further increase in strain results in a decrease in the specimen’s load-bearing capacity, causing the stress-strain curve to transition into a descending phase characterized by a sharp peak. The stress at this peak represents the maximum compressive strength of the concrete prism $\left(f_{c}\right)$, while the corresponding strain is referred to as the peak compressive strain ($\varepsilon_{0})$.

**Figure 7:**
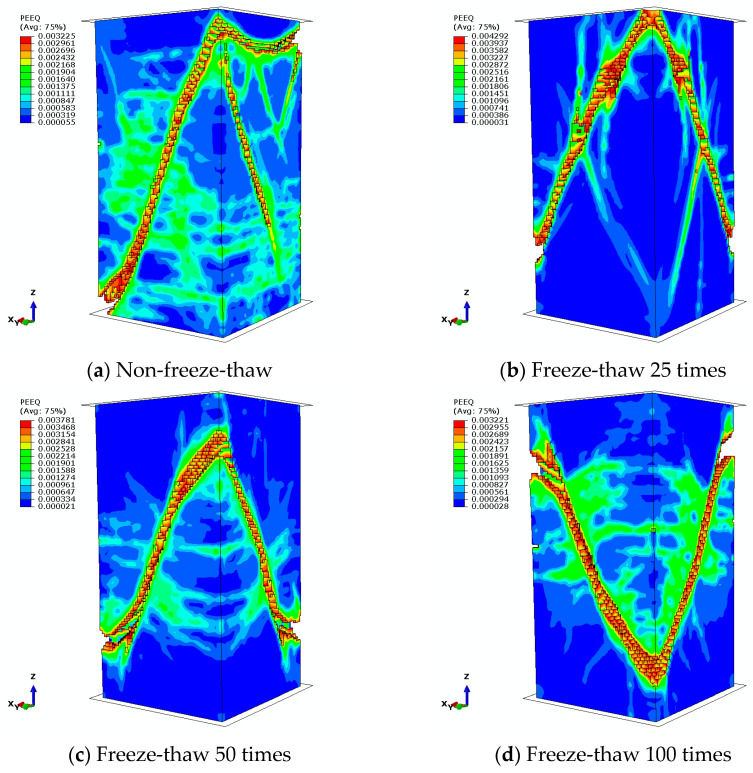
Plastic strain cloud (after damage).

Following the peak load, a gradual reduction in stress was observed, accompanied by the formation of cracks at the interface between the coarse aggregate and the cement mortar within the concrete. As the cracks propagated outward, the first visible crack appeared on the surface of the test block, approximately aligned with the direction of the applied force. With the continued increase in load, multiple discontinuous longitudinal short cracks emerged sequentially on the test block, resulting in a rapid decline in its load-bearing capacity. The interfacial bond between the aggregate and the mortar, as well as the cracks within the mortar itself, continued to propagate and expand. These cracks interconnected, ultimately resulting in the formation of macroscopic diagonal cracks along the weakest plane of the test block, which eventually traversed the entire interface. As the strain on the test block increased, oblique cracks developed and expanded under the influence of both positive stress and shear stress, forming a fractured band, while other regions of the test block exhibited minimal crack development. The plastic strain distribution of the concrete prism post-damage is illustrated in Figure 7, which indicates that a comprehensive diagonal crack ultimately formed throughout the test block following the damage.

**Figure 8:**
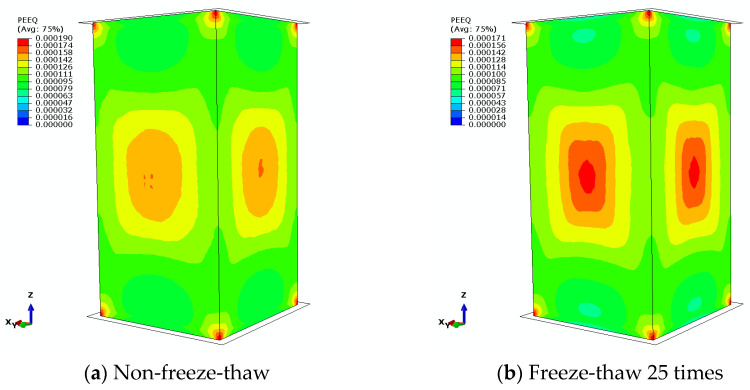

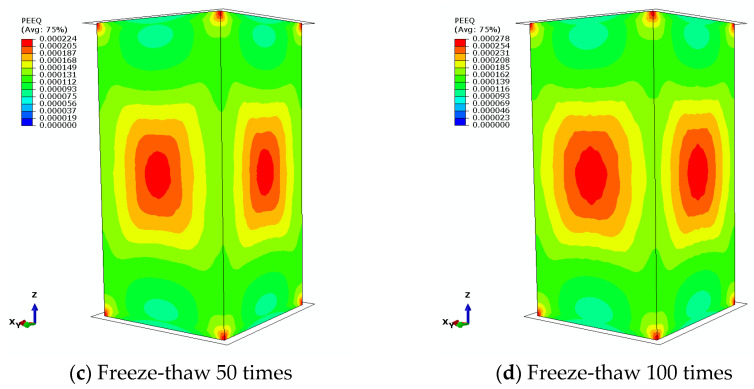
Plastic strain cloud (before damage).

Figure 8 presents the plastic strain cloud diagram of concrete prior to experiencing damage. It is apparent that in the absence of freeze-thaw cycles, the maximum plastic strain is primarily concentrated in the central region of the specimen as well as at the ends of the outer surface. As the number of freeze-thaw cycles increases, the area exhibiting maximum plastic strain in the central region progressively expands until it reaches a specific threshold, beyond which further expansion ceases. Simultaneously, the value of the maximum equivalent plastic strain exhibits a gradual increase corresponding to the rising number of freeze-thaw cycles. This trend signifies a continuous advancement of plastic deformation and damage within the specimen.

### 4.2. Comparative Analysis of Test and Finite Element Method

**Figure 9:**
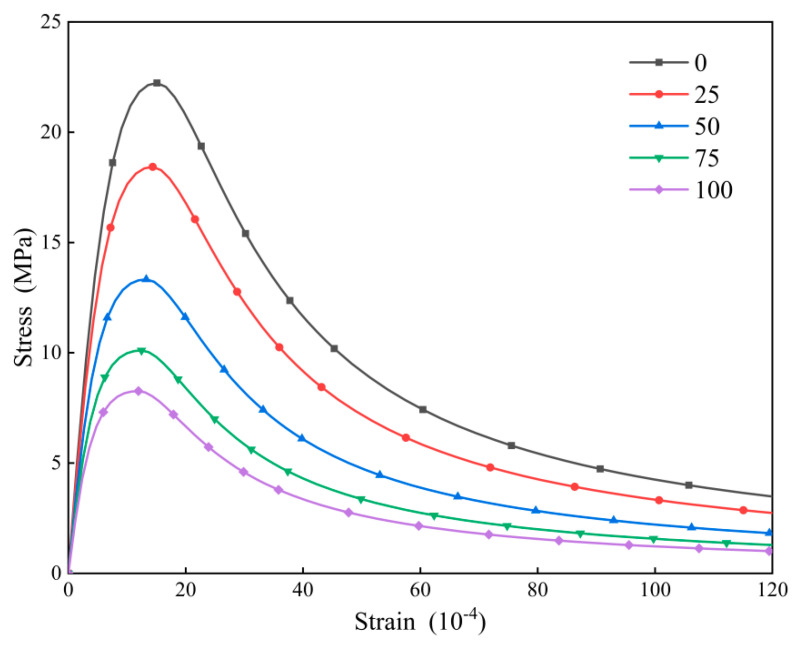
Stress-strain curve at 10^−2^/s strain rate.

The results of the constitutive analysis of concrete subjected to varying numbers of freeze-thaw cycles are presented in the stress-strain curve illustrated in Figure 9. The peak stress and the corresponding compressive strain of the concrete under these conditions are presented in Table 5. It is evident that both peak stress and peak strain exhibit substantial variations under the effect of freeze-thaw cycles. As the number of freeze-thaw cycles increases, the stress-strain curve becomes more gradual. This phenomenon can be ascribed to the progressive deterioration of the internal structure of the concrete specimens. Such degradation results in the development of larger voids and an increase in brittleness, which ultimately culminates in a decrease in strain. This observation aligns with the trends depicted in the previously discussed load-displacement graph.

The data presented in Table 5 demonstrate a reduction in the peak stress of concrete prismatic specimens subjected to uniaxial compression as the number of freeze-thaw cycles increases. Specifically, after 25 freeze-thaw cycles, the peak stress is reduced to 82.9% of its initial value; after 50 cycles, it decreases to 59.9%; following 75 cycles, it further diminishes to 45.5%; and after 100 cycles, it reaches 37.2%. These findings are consistent with the previously established relationship derived from earlier analyses.

In the existing literature, Duan and Qian [27] conducted an investigation into the mechanical characteristics of concrete cubic specimens exhibiting compressive strengths between 30 and 50 MPa following exposure to freeze-thaw cycles. A functional relationship has been established that correlates the relative peak stress, the number of freeze-thaw cycles, and the cubic compressive strength.

(12) $$f_{c}^{D}/f_{c}=1-200\times f_{\mathrm{cu}}^{-3.0355}\times N$$

In this context, $f_{cu}$ is the cubic compressive strength of concrete without freeze-thaw.

In a study conducted by Yang [28], the mechanical characteristics of concrete were investigated, with a particular emphasis on the cubic compressive strength of specimens exhibiting a strength of 33.1 MPa after undergoing freeze-thaw cycles. Furthermore, researchers Shang [29] established a functional relationship between relative peak stress and the number of freeze-thaw cycles, as well as cubic compressive strength, based on tests performed on concrete specimens with an axial compressive strength of 34.2 MPa.

(13) $$f_{c}^{D}/f_{c}=1.00714-0.00453\times N\left(N\geq 2\right)\left(f_{\mathrm{cu}}=33.1\mathrm{MPa}\right)$$

(14) $$f_{c}^{D}/f_{c}=1.0-0.0054308\times N\left(f_{c}=34.2\mathrm{MPa}\right)$$

**Figure 10:**
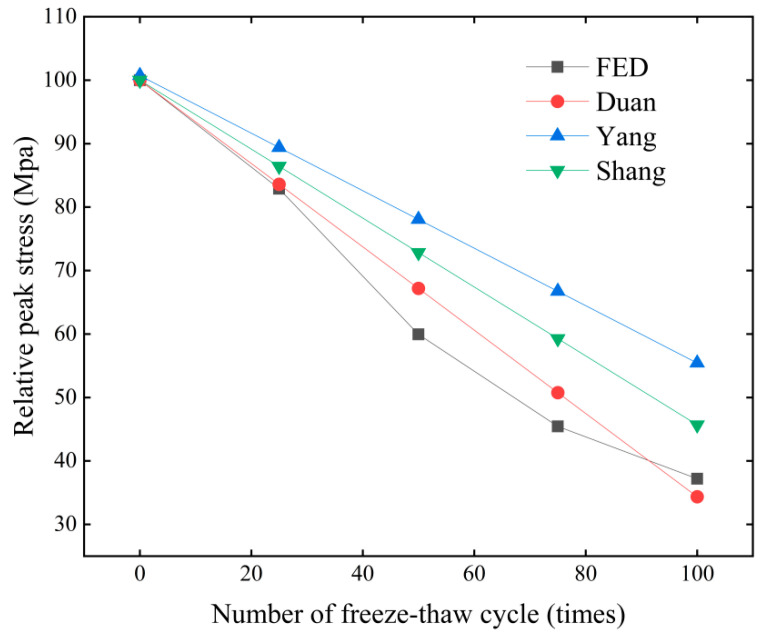
Peak stress versus number of freeze-thaw cycles.

By substituting $f_{cu}$ = 30 MPa into Equation (12), the results of the aforementioned fitting equation, along with the modified constitutive simulation outcomes, are presented in Figure 10. The data obtained from the finite element simulations exhibit a close alignment with the experimental data documented in the literature, particularly in relation to the number of freeze-thaw cycles, with only minimal discrepancies noted. These variations may be attributed to the use of specimens with differing strength grades. Simultaneously, a comprehensive correlation fitting analysis with equal weights of 0.5 was conducted by combining the modified constitutive model with the fitting formula obtained from literature using grey theory. The comprehensive correlation degrees for Duan, Yang, and Shang were 0.867, 0.581, and 0.704, respectively. This indicates that the modified constitutive model in this paper has a high correlation with the experimental data of Duan and Shang. It is important to highlight that the fitted function reported in the literature approximates a linear relationship, which does not accurately represent the actual damage process of concrete. In contrast, this study introduces a theoretical model for concrete damage during freeze-thaw cycles by employing a logistic function, thereby effectively addressing this limitation. Building upon this foundation, a corresponding constitutive model has been developed, yielding simulation results that closely correspond with empirical test outcomes. This model demonstrates an enhanced capability in simulating the mechanical behavior of concrete under freeze-thaw conditions and holds significant potential for application in simulation and predictive analyses within practical engineering projects.

The peak compressive strain is defined as the strain value that corresponds to the maximum stress observed on the complete concrete compressive stress-strain curve. This peak strain serves as an indicator of the deformation capacity of the concrete specimen at the point of maximum destructive load. An analysis of the data presented in Table 5 indicates that the peak strain of concrete prisms under uniaxial compression progressively decreases with an increasing number of freeze-thaw cycles.

In their study, Shang et al. [29] established a functional relationship between the strain at the peak stress point and the number of freeze-thaw cycles experienced by concrete subjected to uniaxial compression. This relationship was derived from tests conducted on concrete specimens exhibiting an axial compressive strength of 34.2 MPa.

(15) $$\varepsilon_{c}^{D}=0.0002693\times N+0.0023376$$

The findings of this study suggest that the equation reveals a trend in which the peak strain increases with a greater number of freeze-thaw cycles. This observation is in contrast to the results obtained in the current research.

In the existing literature, Guo [23] conducted tests on the cubic compressive strength specimens of concrete with a strength of 36.5 MPa and identified the peak strain associated with various freeze-thaw cycles; however, a definitive relationship between these two variables was not established. The data collected in this study indicate that the peak strain decreases as the number of freeze-thaw cycles increases, which is consistent with the conclusions drawn in this research.

Similarly, Zou et al. [30] reported in their study that based on tests of the cubic compressive strength specimens of concrete with a strength of 50 MPa, the peak strain initially decreases and then increases as the number of freeze-thaw cycles rises.

In conclusion, the relationship between the peak strain of concrete subjected to freeze-thaw cycles and the frequency of these cycles warrants further investigation. It is essential to recognize that, at this stage, the study of concrete durability is a multifaceted area of research influenced by a variety of interacting factors. This complexity necessitates comprehensive experimental data and numerical simulations, as well as a combination of detailed and broad analyses to gain a thorough understanding of the damage mechanisms that impact concrete durability.

Moreover, the dynamic mechanical characteristics of concrete are crucial for examining dynamic water pressure and seismic responses; however, the analysis of these properties within concrete structures is inherently complex. This paper provides only a preliminary exploration of the dynamic response of concrete at a strain rate of 10^−2^/s. Therefore, subsequent research endeavors should focus on the synergistic effects of multiple factors as well as the influence of varying strain rates on damage mechanisms. Furthermore, it is imperative that future studies examine the attenuation parameters of material properties within the framework of freeze-thaw cycles. The development of this model was based on a specific type of concrete and a limited range of freeze-thaw conditions. Consequently, its prediction accuracy may be compromised when applied to other types of concrete, especially those with different mix proportions, aggregate types, or additives. In light of these inherent limitations, it is imperative to explore ways to adapt the model to other concrete types and varying environmental conditions. A viable direction for future research involves conducting in-depth experimental studies using a broader range of concrete types and freeze-thaw conditions. Such research will greatly facilitate the development of more universally applicable models, taking into account the variability in material properties and environmental factors more comprehensively.

It is noteworthy to highlight that our research has specifically refined the damage evolution mechanism of concrete under compression. However, when examining the potential seismic damage risk to high concrete dam structures, tensile strength emerges as a pivotal factor that cannot be overlooked. Indeed, tensile strength directly correlates with the seismic response and overall stability of these structures, serving as a core parameter in assessing earthquake damage risk. Furthermore, for bridge structures, secondary stress-induced cracks also deserve attention. These cracks, predominantly tensile or splitting in nature, pose a significant threat to the durability and safety of bridges. Given these considerations, despite the advancements made in our understanding of concrete compression damage, further research is still required to deepen the exploration of tensile damage evolution in concrete under complex environments, particularly freeze-thaw cycles. Such efforts will not only enhance seismic design capabilities for concrete structures but also provide crucial scientific insights for optimizing the maintenance strategies of infrastructure like bridges.

## 5. Conclusions

The sequential coupling thermal stress method was utilized to model the freeze-thaw process of concrete, resulting in the formulation of a dynamic compressive constitutive model for concrete exposed to freeze-thaw cycles. This model was incorporated into finite element software for simulation purposes and subsequently compared with empirical test results, leading to the following conclusions:

1. A dynamic compressive constitutive model for concrete subjected to freeze-thaw cycles was developed by modifying the parameters that govern the progression of compressive damage in accordance with the *Code* [21].
2. The application of a modified constitutive model in conjunction with a damage plasticity model within ABAQUS software, while considering the influence of the damage factor, enabled a more precise simulation of the uniaxial compressive behavior of concrete subjected to freeze-thaw cycles. This methodology provided a dynamic and real-time representation of stress development and the corresponding damage state of concrete throughout the stress process.
3. A comprehensive correlation analysis was performed using grey theory to combine the modified constitutive model with the fitting formulas obtained from the literature. The comprehensive correlation degrees for Duan [27] and Shang [29] were 0.867 and 0.704, respectively. This indicates that our model has a high correlation with the experimental results.
4. This research can be effectively utilized in ABAQUS simulations as well as in a range of concrete-related engineering applications. For instance, in dam design, it could be used to predict the stress distributions and potential failure modes under various loading conditions, aiding in the optimization of dam structure and material selection. Similarly, in bridge design, the model can simulate the dynamic response of the bridge to traffic loads and environmental factors such as wind and temperature variations, thus contributing to the assessment of bridge safety and durability. These applications underscore the practical relevance and versatility of our model in addressing complex engineering challenges.

## Figures and Tables

**Table 1:** Material Parameters. (Quoted from the *Code* [21]).

Material Behavior	Value	Units
Density	2400	kg/m^3^
Specific Heat	960	J/kg·°C
Conductivity	2.94	W/m·K
Expansion Coefficient	1 × 10^−5^	1/°C
Poisson’s Ratio	0.2	—
Dilation Angle	30	—
Eccentricity	0.1	—
fb0/fc0	1.16	—
K	0.6667	—
Viscosity Parameter	1 × 10^−5^	—

**Table 2:** Amplitude value setting for freeze-thaw cycles.

Time (s)	Value
0	10
900	8.5
1800	4.5
2700	0
3600	−3.5
4500	−7
5400	−10.5
6300	−13.5
7200	−16
8100	−20
9000	−16
9900	−11.5
10,800	−6
11,700	−1.5
12,600	5
13,500	9
14,400	10

**Table 3:** Compressive strength of concrete under freeze-thaw cycles.

Serial Number	Number of Freeze-Thaw Cycles	Peak Force (N)	Compressive Strength (MPa)	Relative Compressive Strength (%)
1	0	468,800.28	20.84	100.00%
2	25	389,388.34	17.31	83.06%
3	50	279,772.94	12.43	59.68%
4	75	214,684.53	9.54	45.79%
5	100	173,318.89	7.70	36.97%

**Table 4:** Dynamic modulus of the elasticity of concrete under freeze-thaw cycles.

Serial Number	Number of Freeze-Thaw Cycles	Dynamic Modulus of Elasticity (MPa)	Relative Compressive Strength (%)
1	0	35,721	100.00%
2	25	32,804	91.83%
3	50	29,909	83.73%
4	75	24,494	68.57%
5	100	22,494	62.97%

**Table 5:** Material Parameters.

Number of Freeze-Thaw Cycles	Peak Compressive Stress (MPa)	Peak Compressive Strain
0	22.23	0.001511
25	18.43	0.001438
50	13.32	0.001328
75	10.10	0.001247
100	8.27	0.001194

## Data Availability

The original contributions presented in this study are included in the article. Further inquiries can be directed to the corresponding author.
